# Supplementary figures and images for: Differential risk of cardiovascular complications in patients with type-2 diabetes mellitus in Ghana: A hospital-based cross-sectional study
Source: PLoS One. 2025 Feb 6;20(2):e0302912. doi: 10.1371/journal.pone.0302912 (PMC11801548; doi:10.1371/journal.pone.0302912)

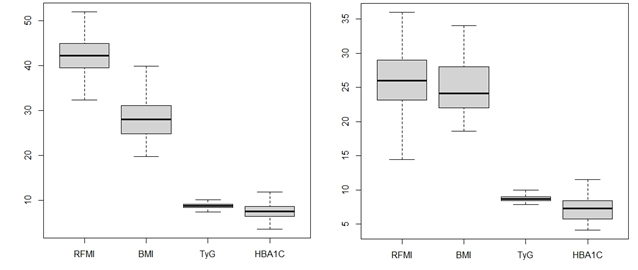

Supplement: S1 Fig — Data for female T2DM (left); data for male T2DM (right). (TIF) [file pone.0302912.s001.tif]

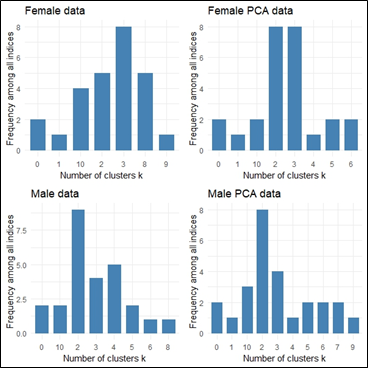

Supplement: S2 Fig — (TIF) [file pone.0302912.s002.tif]

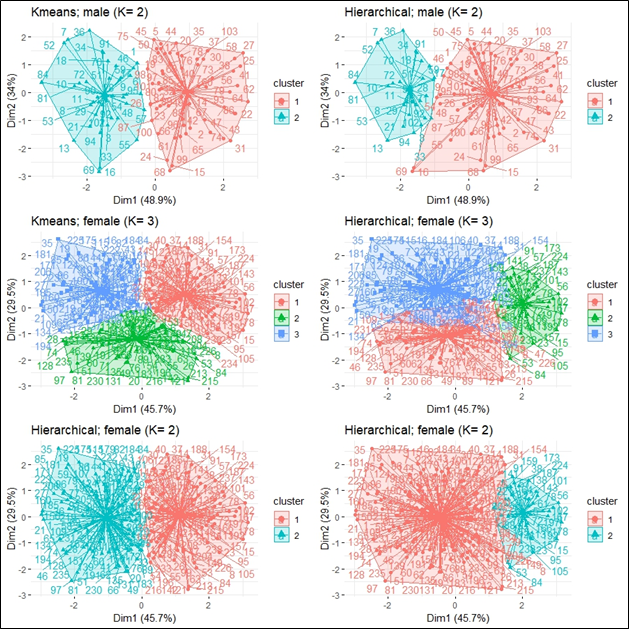

Supplement: S3 Fig — (TIF) [file pone.0302912.s003.tif]
