# Supplementary material for: Differential risk of cardiovascular complications in patients with type-2 diabetes mellitus in Ghana: A hospital-based cross-sectional study
Source: PLoS One. 2025 Feb 6;20(2):e0302912. doi: 10.1371/journal.pone.0302912 (PMC11801548; doi:10.1371/journal.pone.0302912)
Supplement: S1 File — (DOCX) [file pone.0302912.s004.docx]

**Differential risk of cardiovascular complications in patients with adult type-2 diabetes mellitus in Ghana using clustering analysis: A hospital-based cross-sectional study**

***Corresponding Author**

Christian Obirikorang, PhD

Department of Molecular Medicine

School of Medical Sciences

Kwame Nkrumah University of Science and Technology (KNUST)

Kumasi, Ghana

[krisobiri@yahoo.com](mailto:krisobiri@yahoo.com)

**Supplementary data**

# Cluster validation analysis

**Table S1: Internal validation estimates of optimal clusters.**

| **Validation measures** | **Possible Clusters for male data** | **Possible Clusters for female data** | |
| --- | --- | --- | --- |
|  | K=2 | K=2 | K=3 |
| **K-means** |  |  |  |
| Average silhouette score | 0.30 | 0.26 | 0.25 |
| Dunn Index | 0.06 | 0.08 | 0.08 |
| Connectivity score | 32.26 | 80.75 | 115.45 |
| **Hierarchical** |  |  |  |
| Average silhouette width | 0.28 | 0.26 | 0.23 |
| Dunn Index | 0.13 | 0.07 | 0.09 |
| Connectivity score | 21.59 | 33.13 | 92.97 |
| **PAM** |  |  |  |
| Average silhouette width | 0.25 | 0.25 | 0.24 |
| Dunn Index | 0.05 | 0.08 | 0.04 |
| Connectivity score | 35.51 | 94.0 | 138.66 |
